# Novel 3-Hydroxy-2-Naphthoate-Based Task-Specific Ionic Liquids for an Efficient Extraction of Heavy Metals

**DOI:** 10.3389/fchem.2018.00172

**Published:** 2018-05-23

**Authors:** Philip Pirkwieser, José A. López-López, Wolfgang Kandioller, Bernhard K. Keppler, Carlos Moreno, Franz Jirsa

**Affiliations:** ^1^Faculty of Chemistry, Institute of Inorganic Chemistry, University of Vienna, Vienna, Austria; ^2^Department of Analytical Chemistry, Faculty of Marine and Environmental Sciences, University of Cádiz, Puerto Real, Spain; ^3^Department of Zoology, University of Johannesburg, Johannesburg, South Africa

**Keywords:** ionic liquid, ammonium, phosphonium, 3-hydroxy-2-naphthoic acid, heavy metal extraction

## Abstract

Ionic liquids (ILs) are per definition salts with melting points below 100°C and might be green alternatives for the extraction of heavy metals from aqueous solutions due to their favorable environmental and physico-chemical properties. Partial solution during extraction, so-called leaching, however, limits their applicability. The present study synthesizes three novel ammonium and phosphonium ILs based on 3-hydroxy-2-naphthoic acid—trihexyltetradecylphosphonium—([P_66614_]), methyltrioctylphosphonium—([P_1888_]), and methyltrioctylammonium 3-hydroxy-2-naphthoate ([N_1888_][HNA])—by a deprotonation-metathesis route. The aims were to improve stability during extraction while still achieving high selectivity toward heavy metal ions, as well as to study the impact of different alkyl chains and the central atom of the cation on physico-chemical properties, extraction efficacy, and leaching. Extraction capabilities for the seven heavy metals Ag, Cd, Co, Cu, Mn, Ni, and Pb were studied in pure water at pH 8.0. Further experiments were conducted in water containing 30 g L^−1^ NaCl to simulate a seawater matrix and/or 30 mg L^−1^ humic acids, as well as metal-spiked natural water samples. All three ILs showed extraction efficacies ≥90% for Cu and Pb after 24 h. Overall, extraction efficacies for Ag, Cd, Cu, and Pb were highest for drinking water samples. Ag and Cd extraction was increased by up to 41% in (hyper-) saline samples using IL [P_66614_][HNA] compared with pure water samples. Leaching values were reduced down to 0.07% loss of the applied IL, which can be attributed to the hydrophobic character of 3-hydroxy-2-naphthoate. Our results represent a positive development toward a greener extraction of heavy metals from natural waters.

## Introduction

Heavy metals are pollutants of major concern in the aquatic environment globally. They are toxic even at low concentrations, they are persistent and they tend to bio-accumulate (Pacyna et al., 2007; Khan et al., 2008; WHO, 2014). Sources of metal pollution are mainly of anthropogenic origin and include the release of heavy metal-containing wastewaters, the use of contaminated fertilizers in agriculture, the release from contaminated soils in the vicinity of mining sites, metallurgy itself, or emissions during energy production (e.g., coal burning). In addition, metals can enter waterbodies either in aquatic solutions or after atmospheric transport and subsequent dry or wet deposition (Järup, 2003; Nagajyoti et al., 2010). Efforts to reduce pollution in wastewaters and runoff have led to the development of several methods for metal removal and recovery. Amongst these are solvent extraction, adsorption, or precipitation. These methods have, besides their advantages, certain limitations such as lack of selectivity, expensive maintenance or the use of toxic, and/or flammable volatile organic compounds. Precipitation methods, for example, produce great quantities of heavy metal-loaded sludge, whereas ion-exchange and electrochemical methods are too expensive for large volumes of water, and membrane filtration is costly, mainly due to membrane fouling (Kentish and Stevens, 2001; Stojanovic and Keppler, 2012). Therefore, research toward more efficient and environmentally friendly alternatives for extracting metals from aqueous phases, including wastewaters and natural waters, has increased in the last years (Zhao et al., 2005).

A new class of chemical compounds has been discovered as suitable alternatives for traditional chemical treatment of polluted water: Ionic liquids (ILs). Those compounds, consisting solely of ions, have been frequently proposed as more sustainable and environmentally friendly alternatives for several applications such as solvent extraction, separation, organic synthesis, or electrochemistry (Seddon, 1997; Holbrey and Seddon, 1999; Rogers et al., 1999; Earle and Seddon, 2000; Marsh et al., 2002; Wasserscheid et al., 2002; Wei et al., 2003; Kogelnig et al., 2010). ILs exhibit a large spectrum of unique properties, including high thermal stability, liquid states over a wide temperature range, a very low vapor pressure, varying lipophilicity, or recyclability, some of which are sought-after, environmentally favorable characteristics (Visser et al., 2001a; Dietz, 2006; Stojanovic and Keppler, 2012). The synthesis of task-specific ionic liquids (TSILs) has received increasing interest with regard to metal extraction because ILs can be “tailored” to meet the intended, special properties. By introducing metal-chelating functional groups, the affinity of the ILs toward metal ions can be fine-tuned, leading to better efficacies in extraction processes (Visser et al., 2001b; Lee, 2006). Successful metal extraction has been achieved by introducing functional groups in cations as well as anions. These include urea-, thiourea-, or thioether-substituted alkyl groups on the alkyl chains of imidazoles (Visser et al., 2002) as well as (thio-)salicylate (Stojanovic et al., 2010; Leyma et al., 2016), maltolate (Platzer et al., 2015), or thioglycolate (Platzer et al., 2016) groups as part of the anion. By choosing additional properties, e.g., the length of the alkyl chains on the cation, TSILs may simultaneously act as hydrophobic solvents and extractants (Zhao et al., 2005). Kogelnig et al. (2008) proposed a “greener” approach for the synthesis of TSILs. This included searching for higher yields, fewer byproducts and avoiding environmentally harmful chemicals by applying a simple deprotonation and anion metathesis strategy with commercially available precursors. A more recent synthesis route utilizing methylcarbonate precursors was proposed to further improve this strategy because only carbon dioxide and methanol are formed during production, allowing for an even simpler and “greener” reaction in quantitative yield and high purity without further purification (Platzer et al., 2016).

Liquid-liquid extraction of metals from aqueous phases revealed a major drawback regarding green applicability: partial solution of the ILs during extraction, so-called leaching. Even if TSILs display excellent extraction capabilities, strong leaching can challenge their status as an environmentally friendly alternative (Stojanovic and Keppler, 2012).

In the search for TSILs that combine high effectiveness in metal extraction with minimal leaching, this research synthesized novel compounds collating those properties. Accordingly, three different ILs were developed using 3-hydroxy-2-naphthoate as anion and methyltrioctylammonium, methyltrioctlyphosphonium as well as trihexyltetradecyl-phosphonium as the respective cations. After physico-chemical characterization, their extraction efficacy and leaching behavior were studied in different aqueous solutions. These included synthetic matrices of different composition, as well as natural drinking- and seawater, a hypersaline water sample and the effluent of a wastewater treatment plant. We also assessed the impact of structural differences of the ILs, in this case the change of the central atom or the alkyl chain length of the cation, on extraction efficacy and leaching.

This approach yielded further insights into both extraction capabilities as well as leaching behavior and therefore into the environmental and economic merits of “tailored” ILs. Especially the data for different natural water samples present a basis for future research on the potential applicability of the novel ILs in heavy metal removal and recovery from different aqueous matrices.

## Materials and methods

### Solvents and reagents

The IL precursors trihexyltetradecylphosphonium chloride (95%) and 3-hydroxy-2-naphthoic acid (98%) were purchased from Sigma-Aldrich (USA), methyl-trioctylphosphonium methylcarbonate (purum, 50% in methanol) and methyl-trioctylammonium methylcarbonate (purum, 50% in methanol) from Proionic (Austria). For synthesis and sample preparations, potassium hydroxide (p.a., 85%) from Merck (Germany), methanol (HPLC grade, 99.9%), dichloromethane (HPLC grade, 99.9%), sodium chloride (99%), nitric acid (p.a., 65%), and sodium hydroxide (98%) from Panreac (Spain), and humic acid sodium salt (technical grade) from Sigma-Aldrich were used. Standard solutions of metals Ag, Cd, Co, Cu, Mn, Ni, and Pb 1,000 mg L^−1^ in 2–4% (w/w) HNO_3_, purchased from Sigma-Aldrich, were used for atomic absorption spectroscopy measurements and to prepare feed solutions. Ultra-pure water of resistivity <18.2 MΩ cm was obtained from a Millipore Milli-Q Academic apparatus (Merck Millipore, USA).

### Apparatus

Ionic liquids were characterized by spectroscopic analysis. ^1^H NMR spectra were recorded on an Avance III^TM^ 500 MHz spectrometer (Bruker, USA) in CDCl_3_ at 298 K using standard pulse programs at 500.10 MHz (^1^H). FT-IR spectra were measured with a Vertex 70 Fourier transform IR spectrometer (Bruker, USA). Melting points were determined with a Büchi (Switzerland) Melting Point M-560 and density measurements with a Hofmann Klaus calibrated glass pycnometer. Viscosity was measured using a Kinexus rheometer from Malvern Instruments (UK) as plate/plate method (PU20) in a temperature range of 293–323 K with a temperature ramp of 5 K/min, a gap of 1 mm, 4 Pa shear stress, and 20 s^−1^ shear rate. Water content of the ILs was determined using a Mettler Toledo (USA) DL39 Karl Fischer coulometer. Elemental analysis was done using an “ES 1108 CHNS-O” analyzer, and capillary electrophoresis with a conductivity detector (Crystal 310) by Thermo Fisher (USA) was used to measure the chloride content of [P_66614_][HNA]. To determine pH-values a BasiC 20 pH meter was utilized, conductivity of the feed solutions was measured by conductometer BasiC30, and their chloride contents were determined by an ion selective electrode pH and ION meter GLP 22^+^ (Crison, Spain). Samples were stirred with Labbox instruments H01 series magnetic stirrers (Labbox, Spain).

Concentrations of metals in the samples before and after extraction were measured by high-resolution continuum source flame atomic absorption spectrometer contrAA® 700 (Analytik Jena, Germany). The instrument was set up to allow multi-elemental measurement of all used metals, measuring the following wavelengths: Ag 328.07 nm, Cd 228.80 nm, Co 240.73 nm, Cu 324.75 nm, Mn 279.48 nm, Ni 232.00 nm, and Pb 217.00 nm. An acetylene/air flame was utilized and the spectrometer detector was set up between pixels 90–112. In order to evaluate the degree of leaching of the IL into the aqueous phase, the total organic carbon (TOC) in the samples was measured using a multi N/C 3100 analyzer (Analytik Jena, Germany).

### Synthesis of the ionic liquids

The IL trihexyltetradecylphosphonium 3-hydroxy-2-naphthoate ([P_66614_][HNA]) was synthesized by a deprotonation-metathesis reaction, performed as described for other trihexyltetradecylphosphonium-based ILs (Kogelnig et al., 2008; Stojanovic et al., 2010). Specifically, 3-hydroxy-2-naphthoic acid was transferred to a round-bottom flask, dissolved in methanol and a 1.2 equimolar amount of KOH was added for deprotonation of the acid. Afterwards, an equimolar amount of cation precursor trihexyltetradecylphosphonium chloride dissolved in methanol was added to the reaction mixture. The mixture was stirred at 40°C for 3 h. Methanol was then evaporated using a rotary evaporator and the resulting residue was extracted with dichloromethane/water. The separated organic phase was finally dried over anhydrous Na_2_SO_4_, filtered, concentrated under reduced pressure, and dried *in vacuo* overnight. The so prepared IL was stored in a dark glass bottle at room temperature.

For the ILs methyltrioctylphosphonium—([P_1888_][HNA]) and methyltrioctylammonium 3-hydroxy-2-naphthoate ([N_1888_][HNA]), an equimolar amount of the methylcarbonate cation precursors dissolved in methanol was added to the methanol solution of 3-hydroxy-2-naphthoic acid. This solution was stirred for 1 h, methanol was evaporated afterwards and the product was concentrated under reduced pressure, dried *in vacuo* overnight, and stored in a dark glass bottle at room temperature.

### Feed solutions for extraction experiments

Synthetic feed solutions consisted of Milli-Q water (“pure water”), pure water with 30 g L^−1^ NaCl (“water-NaCl”), with 30 mg L^−1^ humic acid sodium salt (“water-HS”), and a combination of both (“water-HS-NaCl”). All synthetic feed solutions were adjusted to an initial pH of 8.0, which represents the average pH of natural saline water in the sampling region. The natural water samples were taken from the following sites in the Bay of Cádiz, southwest Spain: Río San Pedro in Puerto Real, which is an arm-of-the sea of the Bay of Cádiz (“seawater”), the effluent of a wastewater treatment plant (“WWTP effluent”) in Puerto Real, drinking water from the University of Cádiz Campus Puerto Real (“drinking water”), as well as a hypersaline sample collected from the salt works company “Salinas Las Tapas” in El Puerto de Santa María (“hypersaline water”). For the composition of the respective feed solutions used in extraction experiments see Table 1.

**Table 1:** Composition of feed solutions used for extraction experiments.

	**pH**	**NaCl (g L^−1^)**	**Conductivity (mS cm^−1^)**	**DOC (mg L^−1^)**	**Added humic acid salt (mg L^−1^)**
**SYNTHETIC FEED SOLUTIONS**					
Pure water	8.0	–	<0.01	<0.5	–
Water-NaCl	8.0	30.0	38.3	<0.5	–
Water-HS	8.0	–	0.032	11.0	30.0
Water-HS-NaCl	8.0	30.0	38.4	11.0	30.0
**NATURAL FEED SOLUTIONS**					
Drinking water	7.82	0.04	0.552	1.1	–
WWTP effluent	8.27	0.47	1.76	14.0	–
Seawater	8.02	36.32	43.0	1.6	–
Hypersaline water	7.91	60.51	54.0	7.1	–

All feed solutions were spiked with 1 mg L^−1^ Ag, Cd, Co, Cu, Mn, and Ni as well as 4 mg L^−1^ Pb. The natural water feed solutions were filtered using 0.45 μm nylon microfiltration membranes (Dorsan, Spain) before spiking. Measured parameters of natural drinking water were well-comparable to data given by the Spanish authorities (CAZG, 2017).

### Extraction experiments

In order to evaluate the potential of the synthesized ILs for metal extraction, 300 mg of IL were weighed into a polyethylene bottle and 30 mL of the respective feed solution were added. The two ILs which are solid at room temperature, [P_1888_][HNA] and [N_1888_][HNA], were brought just above melting point in a hot water bath before transferring them into the PE bottle. Feed solution was added after their subsequent solidification at room temperature. The ILs remained solid during extraction, resulting in a solid-liquid extraction in these two cases. The samples were kept at room temperature and were stirred at 300 rpm during extraction. Afterwards, the aqueous phase which was used for measurement was recovered by pipetting without further separation. Experiments were done in triplicates. To consider a loss of metal due to possible oxidation-, precipitation-, and adhesion effects in the tubes, reference samples containing the respective metal concentrations without IL were treated similar to the samples with ILs and the metal concentration was determined before and after the respective extraction time. First experiments were conducted with “pure water” over a period of 24 h to obtain a first overview of the total extraction capacity over time. Subsamples were measured for their metal content after 1, 2, 4, and 24 h. For [P_66614_][HNA] an additional subsample was measured after 8 h.

To investigate the effect of different sample compositions on extraction and leaching, an extraction time of 1 h was chosen, as the extraction efficacies in “pure water” samples after 1 h were high enough as to easily assess differences.

### Quantification

The extraction efficacy was calculated as the percentage of metal removed from the feed solution after the extraction experiment using the following Equation (1).

(1) $$\begin{array}{l} Extraction\;efficacy\;\left(\%\right)=\frac{C_{Ref}\;-\;C_{t}}{C_{Ref}}\;*100 \end{array}$$

*C*_*Ref*_ represents the metal concentration in the reference after the respective extraction time and *C*_*t*_ the metal concentration in the sample after extraction.

To evaluate the degree of leaching, the total organic carbon concentration was measured in samples after extraction (Equation 2). For “water-HS,” “water-HS-NaCl” and natural water samples, the TOC was measured before extraction and subtracted from the value after extraction.

(2) $$\begin{array}{l} Leaching\;\left(\%\right)=\frac{\Delta TOC^{*}V_{S}}{m_{IL}}*100*C_{IL} \end{array}$$

ΔTOC (mg L^−1^) was the measured concentration of total organic carbon in the sample after extraction, minus the measured TOC already present in the sample before extraction, *V*_*s*_ (L) the feed solution volume and *m*_IL_ (mg) the mass of IL used. *C*_IL_ represents the carbon content of the respective IL and was used to calculate the leaching of the entire liquid based on the carbon leaching, on the assumption that the cation and anion of the IL leach equally. The calculated value therefore represents the percental loss of IL during extraction.

## Results and discussion

### Synthesis and characterization of ILs

The structures of the obtained ILs are presented in Table 2. The selected anion, 3-hydroxy-2-naphthoate, remained the same for all three synthesized ILs, whereas the composition of the cation was changed minimally. Especially ILs [P_1888_][HNA] and [N_1888_][HNA] differ only in the quaternary elements, while the alkyl chains are identical. This enables a direct comparison of the impact of the central atom on chelating properties between otherwise identical ILs. 3-Hydroxy-2-napthoate was chosen as the anion because it contains a salicylate functional group which is known for its complexation potential (Fischer et al., 2011), whereas its naphthalene backbone provides the needed hydrophobicity together with the respective cation. The respective synthesis route and characterization of the novel ILs is presented in the following.

**Table 2:** Structures and physico-chemical properties of the synthesized ILs.

**Ionic liquid**	**Structure**	**Physical state at room temperature**	**Melting point**	**Density^a^**	**Water content**	**Viscosity^a^**	**Chloride content**
			**(°C)**	**(g cm^−3^)**	**(%)**	**(cP)**	**(wt%)**
[P_66614_][HNA]	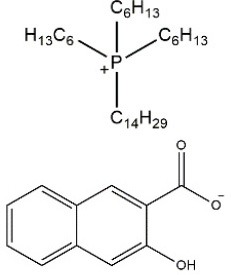	Dark orange viscous oil	n/a	0.97	0.43	714	0.15
[P_1888_][HNA]	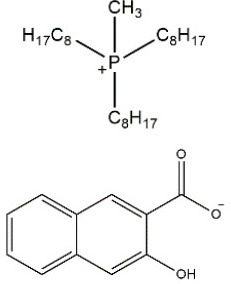	Orange solid	65	0.91	0.32	n/a	n/a
[N_1888_][HNA]	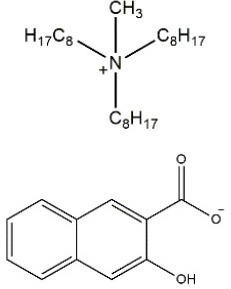	Yellow-orange solid	77	0.87	0.36	n/a	n/a

a *measured at 25°C. n/a, not applicable*.

#### Synthesis of [P_66614_][HNA] and spectra evaluation

IL [P_66614_][HNA] was synthesized via a standard deprotonation-metathesis reaction (Kogelnig et al., 2008; Stojanovic et al., 2010). The use of a chlorinated precursor and the subsequent formation of potassium chloride required purification steps in the form of an extraction with dichloromethane/water, drying over anhydrous Na_2_SO_4_, and filtration. The IL was obtained in a high yield (≥95%) after the washing steps. A low chloride content of 0.15 wt% was achieved (Table S1), which is in line with or significantly lower than the values of similarly synthesized TSILs from chlorinated precursors, with contents up to 6 wt% (Mikkola et al., 2006; Kogelnig et al., 2008; Stojanovic et al., 2010; Fischer et al., 2011; Leyma et al., 2016). The structure of the IL was analyzed by evaluating FT-IR, ^1^H-NMR as well as MS spectra, confirming the desired constitution of the compound.

Regarding the anion, FT-IR confirms the deprotonation of the acid based on the absence of a broad –OH absorption band from the carboxyl group. Bands in the region of 1,600 cm^−1^ indicate a carboxylate group as well as aromatic C = C double bonds. The ^1^H-NMR spectrum (Figure S1) shows characteristic shifts for aromatic hydrogen atoms between 7.75 and 7.14 ppm. Regarding mass spectra, the C_11_H_7_O_3_ peak at 187 m/z confirms the presence of 3-hydroxy-2-naphthoate.

For the cation trihexyltetradecylphosphonium, the FT-IR spectrum shows bands at 2,925 and 2,857 cm^−1^, which indicate aliphatic –CH_2_ asymmetric stretching bonds. The ^1^H-NMR spectrum shows a peak between 0.92 and 0.77 ppm, which can be attributed to the 12 protons found in the four terminal CH_3_-groups. The remaining protons of –CH_2_-groups are split up due to shifts caused by the phosphonium group, with a peak between 2.32 and 2.17 ppm belonging to the eight protons in the primary –CH_2_-groups of the alkyl chains. The mass spectrum shows a peak at 483.7 m/z, which corresponds to the structure of the cation trihexyltetradecylphosphonium, C_32_H_68_P.

#### Synthesis of [P_1888_][HNA] and [N_1888_][HNA] and spectra evaluation

Methyltrioctylphosphonium- and methyltrioctylammonium 3-hydroxy-2-naphthoate were synthesized using methylcarbonate precursors, as received from the manufacturer dissolved in methanol. During reaction, only carbon dioxide and methanol were formed as side products, allowing a simple, straightforward, and green synthesis. The presented synthesis route reduces the possibility of inorganic impurities, renders following clean-up steps unnecessary, and leads to quantitative yields. This fast, quantitative reaction without use of additional solvents, reagents or clean-up steps is in line with principles of green chemistry (Anastas and Warner, 1998).

Regarding the anion, both ILs show the characteristic peaks in all spectra as found for IL [P_66614_][HNA] and shown above. Concerning the cations, differences due to the central atom, which presents the only difference between the two ILs, are visible in the ^1^H-NMR spectra (Figures S2 and S3). A larger shift caused by ammonium for [N_1888_][HNA] is evident for the three protons of the methyl group at 3.18 ppm and the six protons of –CH_2_-groups at 3.34–3.22 ppm, as opposed to the same, smaller shifts caused by phosphonium for [P_1888_][HNA] at 1.95 and 2.27–2.14 ppm, respectively. Additionally, we recorded MS peaks at 368.4 m/z for methyltrioctylammonium formula C_25_H_54_N and 385.4 m/z for methyltrioctylphosphonium formula C_25_H_54_P.

#### Physico-chemical properties of the novel ILs

Physico-chemical properties of the three ILs are presented in Table 2. Alkyl chain lengths and the central atom of the cation strongly affect the melting point and therefore the physical state at room temperature. As previously observed for similar structures, larger cations tend to decrease the melting point (Platzer et al., 2016). This is the case for the cation trihexyltetradecylphosphonium of [P_66614_][HNA], which is the only substance of the three that was liquid at room temperature. ILs [P_1888_][HNA] and [N_1888_][HNA] have a shorter cationic residue and are solid. This is also in line with observations that methyltrioctylammonium-based ILs are more likely to be solid at room temperature when compared to ILs based on trihexyltetradecylphosphonium chloride (Stojanovic et al., 2010).

Differences in physico-chemical properties due to the different quaternary elements nitrogen and phosphorus for instance are reflected in a 12°C lower melting point for [P_1888_][HNA] vs. [N_1888_][HNA]. This difference can be explained by the stronger charge-delocalization of N-containing ILs, leading to higher polarity and to stronger cation–anion interactions, therefore yielding a more compact structure (Carvalho et al., 2014). Density values are <1 g cm^−3^ and in agreement with reported data from previously synthesized ammonium and phosphonium ILs (Bradaric et al., 2003; Stojanovic et al., 2010). Also in line with previously described, similar TSILs, the stronger cation–anion interaction does not lead to the proposed increased density for the ammonium based IL (Platzer et al., 2016). The observed tendency of increased density with bulky, aromatic anions is evident in [P_66614_][HNA], with a value of 0.97 g cm^−3^ compared to 0.93 g cm^−3^ using the smaller salicylate as the anion (Stojanovic et al., 2010). Water contents likewise match or are significantly lower than published data, with values between 0.32 and 0.49%, as is also the case for the viscosity value of 714 cP for [P_66614_][HNA] (Figure S4). As was the case with density, this value is slightly increased compared to the 567 cP when utilizing the less bulky salicylate anion (Stojanovic et al., 2010). Furthermore, all three ILs in this study are miscible with common polar organic solvents such as ethanol, acetone, acetonitrile, or ethylacetate (Table S2). Less polarity of phosphonium ILs also leads to miscibility with diethyl ether.

### Metal extraction

In order to evaluate the applicability in the extraction of metals, the extraction efficacy of the three synthesized ILs for Ag, Cd, Co, Cu, Mn, Ni, and Pb was first studied in synthetic water samples that differed in ionic strength and chelating properties (dissolved organic matter). To gain a first insight in the possible extraction behavior under natural conditions, metal-spiked natural water samples were used for further experiments.

#### Extraction capability of [P_66614_][HNA] in synthetic water samples

**Figure 1:**
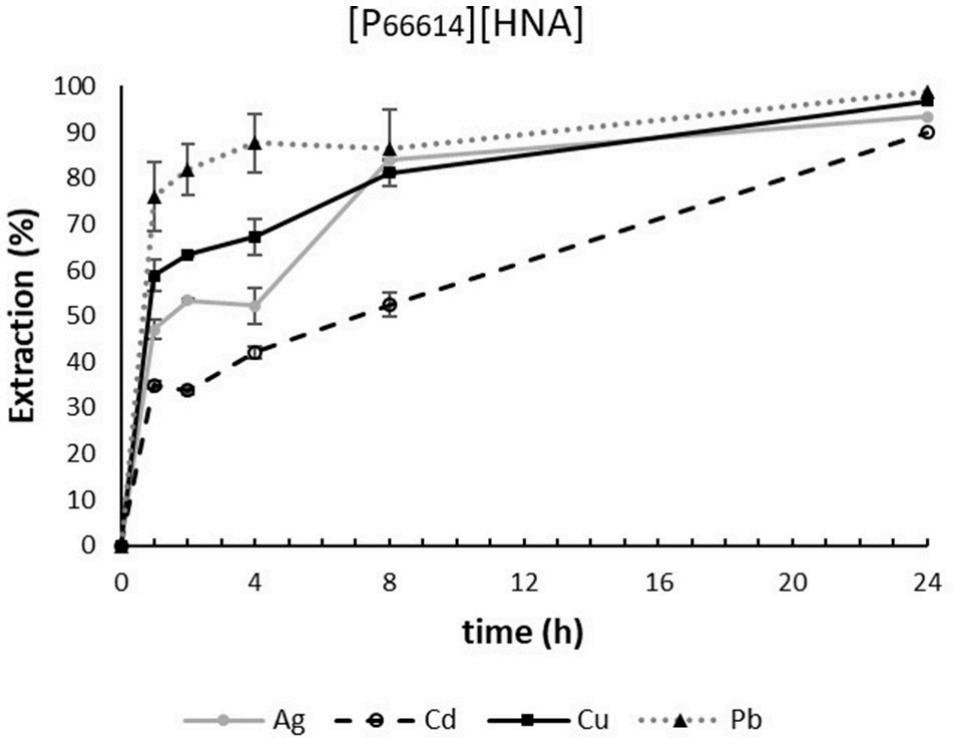
Time dependency of extraction using 300 mg of [P_66614_][HNA] in 30 mL “pure water” feed solution. n = 3, error bars = ± SD.

[P_66614_][HNA] achieved extraction efficacies ≥90% for Ag, Cd, Cu, and Pb after 24 h. As visible in the time profile (Figure 1), Pb extraction already stabilized after 4 h; for all other metals, extraction efficacy increased until 8 h. Also, after the first hour, a clear order of extraction efficacy is evident: Pb (76%) > Cu (59%) > Ag (47%) > Cd (35%). Extraction of Co, Mn, and Ni was lower than 10% and therefore not discussed further.

**Figure 2:**
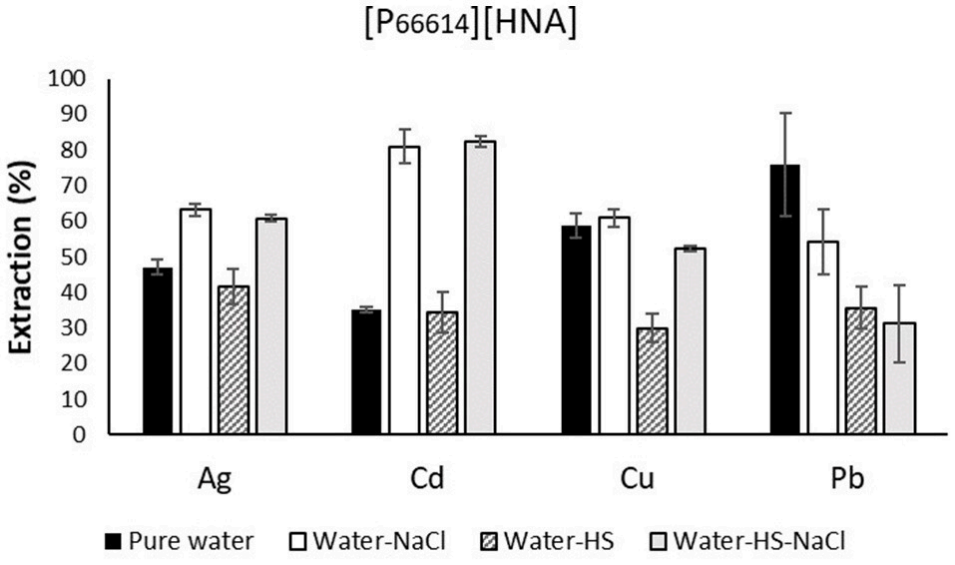
Extraction efficacies in synthetic water samples using 300 mg of [P_66614_][HNA] in 30 mL of the respective feed solution for an extraction time of 1 h. n = 3, error bars = ± SD.

The effects of adding sodium chloride as well as humic acid salt to the samples notably affected the extraction efficacy and are depicted in Figure 2 for a 1-h extraction duration. Generally, the extraction of metals for ILs relies on the mechanisms of ion exchange and neutral extraction, with the balance between the two or the dominance of either one mechanism being highly dependent on sample composition (Janssen et al., 2015). The extraction of Cd, and to a lesser extent also of Ag, was significantly increased when NaCl was present in the sample. Extraction efficacy increased from 35.0 to 80.9% after 1 h for Cd and from 47.1 to 63.3% for Ag. The presence of humic acids did not affect extraction efficacy. Higher extraction efficacies when NaCl was present at a concentration comparable to natural seawater might be explained by the speciation of these metals under such conditions. The dominant species here are chlorido complexes, namely ${\text{CdCl}}_{3}^{-}$, ${\text{AgCl}}_{2}^{-}$, and ${\text{AgCl}}_{3}^{2-}$ (Savenko and Tagirov, 1996; Bhatluri et al., 2014). These species have been described as favoring the anion transfer mechanism of methyltrioctylammonium chloride for IL/seawater or IL/wastewater systems (Altin et al., 2011; Bhatluri et al., 2014; Herce-Sesa et al., 2017a,b). Chloride is present as an impurity in [P_66614_][HNA]. We therefore suggest a similar effect as in the explanation for the observed enhancement of extraction under saline conditions, although this has not been described for phosphonium-based ILs before. For the remaining metals, most notable is a strong decrease in extraction efficacy for Cu and Pb in the presence of humic acids. One potential explanation is the high affinity of both metals toward complexation with humic acids (Kerndorff and Schnitzer, 1980; Liu and Gonzalez, 2000), forming stronger complexes with the latter compared to ILs. Pb extraction decreased in “water-HS-NaCl” samples from 75.9 to 31.3% compared to “pure water” samples. In contrast, Cu extraction was decreased solely with humic acids, from 58.8 to 29.9%; the presence of NaCl almost completely negated this effect and increased the extraction to 52.3%. We explain this by the reduced complexation of Cu in HS due to the formation of CuCl^+^ and the subsequent easier extraction of this species (Lores and Pennock, 1998).

#### Extraction capability of [P_1888_][HNA] and [N_1888_][HNA] in synthetic water samples

**Figure 3:**
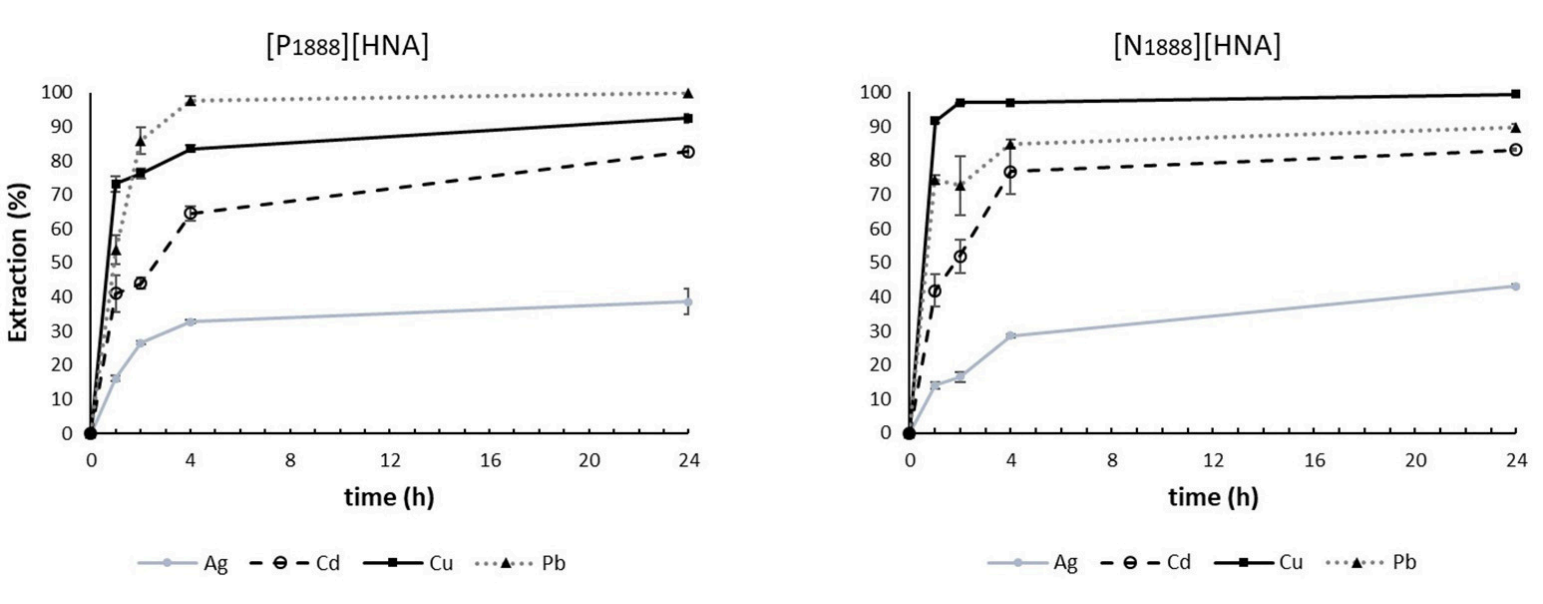
Time dependency of extractions using 300 mg of [P_1888_][HNA] or [N_1888_][HNA] in 30 mL “pure water” feed solution. n = 3, error bars = ± SD.

Both ILs extracted all seven metals out of “pure water” samples but with highly different efficacies (Figure 3). [N_1888_][HNA] generally showed faster extraction than [P_1888_][HNA]. For [N_1888_][HNA], extraction stabilized after 1 h, except for Ag and Cd, where stabilization took longer. The best extracted metal was Cu, with an efficacy of 91.6% after 1 h, followed by Pb (74.4%) and Cd (41.9%). For Cu, a quantitative extraction (>99%) was achieved after 2 h. Using [P_1888_][HNA], the time profile of extraction efficacy shows a stabilization in extraction for all metals after 4 h. The best extracted metal was Pb with an efficacy of 85.9% after 2 h, followed by Cu (76.3%) and Cd (44.1%). Pb could be extracted quantitatively (>95%) after 4 h. We attribute faster extraction using the ammonium IL to the higher polarity of the compound compared to phosphonium IL. Cd extraction was rather slow compared to Cu and Pb using both ILs, but reached a value >80% in both setups after 24 h. Slow but efficient extraction was also observed using [P_66614_][HNA]. This suggests a strong dependence of the extraction mechanism for Cd on the functional group of the anion rather than on cation structure.

Silver was extracted up to approx. 40% with both ILs, as opposed to the efficient extraction (>90%) using [P_66614_][HNA], which has a significantly lower viscosity compared to the other two. Although the reasons for the different extraction remain unclear, our results are in accordance with works of Papaiconomou et al. (2007) and Stojanovic et al. (2010), where extraction efficacy was negatively correlated with the viscosity of the used extractant for Pd and Pt. Although Co, Mn, and Ni were extracted to some degree by [P_1888_][HNA] and [N_1888_][HNA], extraction efficacies surpassing 65% after 24 h could not be achieved in any setup. For that reason, these metals are not further discussed here.

When NaCl and humic acid salt were added to synthetic samples (Table 3 and Figure S5), extraction efficacy for Cu and Pb decreased as discussed for [P_66614_][HNA]. Ag extraction slightly increased in the presence of NaCl for both ILs, but efficacy only decreased for [N_1888_][HNA] in HS samples. Cd extraction likewise did not show the strong increase in efficacy in NaCl samples as observed for [P_66614_][HNA]. These results suggest that there was no efficient anion exchange mechanism of the respective chlorido species in these setups, which can be explained by the absence of Cl^−^ in both ILs.

**Table 3:** Extraction efficacies of the synthesized ILs in spiked synthetic as well as natural water feed solutions for an extraction time of 1 h, n = 3.

		**Extraction efficacy (%)**			
**Sample**	**IL**	**Ag**	**Cd**	**Cu**	**Pb**
Pure water	[P_66614_][HNA]	47.1 ± 2.0	35.0 ± 0.8	58.8 ± 3.5	75.9 ± 7.5
	[P_1888_][HNA]	16.1 ± 0.9	41.1 ± 5.4	73.2 ± 2.3	53.8 ± 4.2
	[N_1888_][HNA]	14.1 ± 0.9	41.9 ± 4.7	91.6 ± 0.7	74.4 ± 1.2
	[P_66614_][HNA]	63.3 ± 1.7	80.9 ± 4.8	60.9 ± 2.4	54.2 ± 9.2
Water-NaCl	[P_1888_][HNA]	24.0 ± 1.6	34.8 ± 3.9	67.7 ± 1.9	43.9 ± 2.8
	[N_1888_][HNA]	37.9 ± 3.3	41.9 ± 7.4	51.5 ± 5.7	40.1 ± 3.7
	[P_66614_][HNA]	41.6 ± 4.8	34.4 ± 5.6	29.9 ± 3.9	35.7 ± 5.8
Water-HS	[P_1888_][HNA]	21.3 ± 0.6	33.0 ± 1.0	22.1 ± 0.5	21.9 ± 4.4
	[N_1888_][HNA]	13.2 ± 4.1	22.8 ± 3.3	20.5 ± 1.6	13.9 ± 7.2
	[P_66614_][HNA]	60.8 ± 1.0	82.4 ± 1.5	52.3 ± 0.7	31.3 ± 10.8
Water-HS-NaCl	[P_1888_][HNA]	32.6 ± 1.7	23.1 ± 3.7	24.9 ± 3.0	23.4 ± 0.9
	[N_1888_][HNA]	8.4 ± 3.7	38.9 ± 5.3	21.7 ± 7.5	22.7 ± 6.3
	[P_66614_][HNA]	81.1 ± 1.5	73.5 ± 6.8	81.3 ± 6.8	75.3 ± 1.4
Drinking water	[P_1888_][HNA]	73.0 ± 3.8	44.0 ± 5.8	71.7 ± 2.3	57.7 ± 4.2
	[N_1888_][HNA]	78.4 ± 8.4	45.2 ± 5.1	91.4 ± 2.3	63.6 ± 6.9
	[P_66614_][HNA]	1.9 ± 1.6	64.4 ± 2.0	51.4 ± 0.0	36.1 ± 2.4
WWTP effluent	[P_1888_][HNA]	0.8 ± 0.5	36.7 ± 1.8	44.6 ± 1.4	20.7 ± 0.0
	[N_1888_][HNA]	3.4 ± 0.5	20.3 ± 6.3	42.2 ± 8.9	6.9 ± 0.0
	[P_66614_][HNA]	62.0 ± 0.9	73.9 ± 0.5	57.4 ± 4.6	39.4 ± 5.3
Seawater	[P_1888_][HNA]	30.7 ± 5.1	56.6 ± 4.6	63.5 ± 6.6	28.3 ± 2.1
	[N_1888_][HNA]	29.0 ± 2.8	52.0 ± 4.0	48.1 ± 5.9	19.3 ± 2.1
	[P_66614_][HNA]	56.9 ± 2.3	76.0 ± 4.4	51.8 ± 9.9	29.5 ± 4.0
Hypersaline water	[P_1888_][HNA]	19.6 ± 5.0	44.0 ± 6.0	31.3 ± 5.9	14.0 ± 2.0
	[N_1888_][HNA]	7.1 ± 0.0	38.3 ± 3.4	29.4 ± 3.3	15.4 ± 2.0

In summary, the three ILs synthesized in this work showed high efficacies toward free divalent metal ions in pure water samples, in particular for Cu, Pb, and Cd. These results also indicate a high capacity of the novel ILs for metal extraction, as the high efficacies were achieved in a multi-elemental setup as opposed to single metal experiments. We conclude that ion exchange of chlorido complexes of Ag and Cd increases extraction efficacy in saline samples for [P_66614_][HNA], whereas complexation with organic carbon led to strong decreases in extraction for Cu and Pb. Generally, the presence of organic matter affected extraction using ammonium-containing IL [N_1888_][HNA] to a higher degree compared to phosphonium ILs. Accordingly, the lower polarity of phosphonium ILs could facilitate ion exchange with the HS-complex, whereas it is hindered between ammonium IL and the HS-complex.

### Leaching

**Figure 4:**
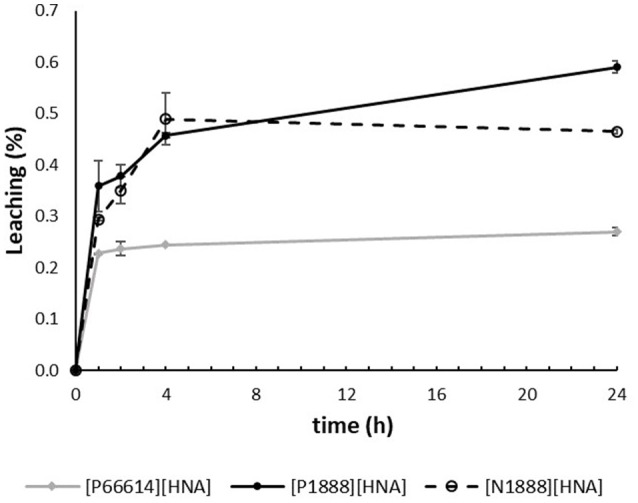
Time dependency of leaching in %, using 300 mg of the respective IL in 30 mL “pure water” feed solution. n = 3, error bars = ± SD.

The leaching behavior of the three synthesized ILs in synthetic water samples was investigated in addition to their extraction capabilities. Leaching over time is depicted in Figure 4. The lowest values were obtained for [P_66614_][HNA], which can be explained by the high carbon-containing alkyl chains and the resulting high hydrophobicity of the cation. The results lie between 0.23% (17.4 mg L^−1^ TOC) after 1 h and 0.27% (20.8 mg L^−1^ TOC) after 24 h, showing a stabilized system after 1 h. Comparing the effect of the central atom on leaching behavior, the ammonium cation of [N_1888_][HNA] was slightly more stable than its phosphonium counterpart. Values of [N_1888_][HNA] ranged from 0.20% (22.6 mg L^−1^ TOC) after 1 h to 0.47% (36.9 mg L^−1^ TOC) after 24 h, stabilizing after 4 h. [P_1888_][HNA] showed an increase in leaching from 0.36% (26.9 mg L^−1^ TOC) after 1 h to 0.59% (44.5 mg L^−1^ TOC) after 24 h. The increased stability of the IL containing a nitrogen central atom agrees with published results on ammonium- and phosphonium-based TSILs with identical anions (Platzer et al., 2016).

Leaching data of NaCl and HS samples (Table 4) showed an increased stability of [P_66614_][HNA] compared with the results for “pure water” samples. A “salting-out” effect due to the higher ionic strength of saline samples, which lowers the solubility of the ILs, might explain this observation (Herce-Sesa et al., 2017b). The lowest value was obtained in the “water-HS-NaCl” samples, with a leaching of 0.13% (9.2 mg L^−1^ TOC). Compared to [P_66614_][HNA], leaching was not significantly reduced for [P_1888_][HNA] and [N_1888_][HNA] in the presence of NaCl, even when humic acids were present in the sample as well. This again points to a different extraction mechanism for [P_66614_][HNA] vs. [P_1888_][HNA] and [N_1888_][HNA], which also potentially influences the leaching behavior of the latter.

**Table 4:** Leaching of the synthesized ILs during extraction in spiked synthetic as well as natural water feed solutions for an extraction time of 1 h, n = 3.

**Sample**	**Leaching %** ±**SD**		
	**[P_66614_][HNA]**	**[P_1888_][HNA]**	**[N_1888_][HNA]**
Pure water	0.227 ± 0.001	0.359 ± 0.049	0.294 ± 0.003
Water-NaCl	0.184 ± 0.003	0.412 ± 0.008	0.355 ± 0.054
Water-HS	0.183 ± 0.004	0.297 ± 0.073	0.261 ± 0.057
Water-HS-NaCl	0.131 ± 0.018	0.411 ± 0.020	0.310 ± 0.050
Drinking water	0.223 ± 0.023	0.405 ± 0.027	0.416 ± 0.123
WWTP effluent	0.068 ± 0.014	0.221 ± 0.024	0.239 ± 0.024
Seawater	0.171 ± 0.006	0.464 ± 0.038	0.468 ± 0.068
Hypersaline water	0.156 ± 0.008	0.341 ± 0.058	0.297 ± 0.032

In summary, the increased hydrophobicity due to the C_14_ alkyl chain of [P_66614_][HNA] led to the lowest leaching in this study. Directly comparing the central atoms, the ammonium IL was more stable than phosphonium during extraction. The focus of most studies dealing with ILs as extractants for metals lies on their extraction capabilities and to a much lesser degree on their leaching behavior. This reduces the possibilities for comparisons. Nevertheless, our data clearly demonstrate that 3-hydroxy-2-naphthoate as a highly hydrophobic anion does strongly reduce leaching compared to the works of Leyma and Platzer (Platzer et al., 2015; Leyma et al., 2016), who investigated ILs composed of similar cations but less hydrophobic anions.

### Application in natural water samples

After investigating the potential to extract heavy metals and the influence of salinity and dissolved organic matter on extraction efficacy and leaching, extraction experiments using metal-spiked natural water samples were carried out. The extraction efficacies of these experiments are summarized in Table 3.

In general, the effects of natural matrices in samples on extraction efficacy agree with the data obtained from synthetic water samples: the influences were similar. The best extraction was achieved for Ag, Cd, Cu, and Pb.

Ag was extracted most effectively out of drinking water by all three ILs, with efficacies between 73 and 81% after 1 h, showcasing a suitability for application in freshwater. The extraction from seawater and hypersaline water was successful using [P_66614_][HNA], showing extraction rates of 62.0 and 56.9%, respectively. These rates agree perfectly with the 63.3% achieved in synthetic NaCl samples. The extraction from the “WWTP effluent” sample was completely inhibited (efficacy <5%), which is most probably due to a high dissolved organic matter content, the presence of other competing ions and low salinity.

Cd extraction was most successful with [P_66614_][HNA] for all samples, including the WWTP effluent, with efficacies ranging between 64 and 76% after 1 h compared to 35% for the “pure water” sample. Comparable to the data of synthetic samples, we explain this increase by the efficient extraction of chlorido species through the proposed anion exchange mechanism. This effect prevailed even under hypersaline conditions with the highest obtained efficacy of 76.0%. In contrast, ILs [P_1888_][HNA] and [N_1888_][HNA] showed only small changes compared with the data from synthetic water samples.

The extraction efficacies of Cu in drinking water after 1 h for [P_1888_][HNA] (71.7%) and [N_1888_][HNA] (91.4%) are similar to the high values achieved in “pure water” samples. In the case of [P_66614_][HNA], Cu extraction even increased from 58.8 to 81.3%. For the remaining samples, efficacies generally were reduced comparable to the decreases recorded in synthetic water samples, depending on the organic carbon content of the sample.

Finally, Pb was extracted equally well from drinking water as from “pure water” samples, with efficacies of the three ILs ranging from 57.7 to 75.3%. Apart from drinking water, Pb extraction was affected the most by sample composition. Extraction efficacies decreased for all remaining samples, similar to synthetic water samples, due to NaCl and organic carbon content. The overall lowest values for Pb were obtained for [N_1888_][HNA], confirming that the effect of the sample matrix on extraction is strongest for the ammonium IL.

Leaching values also were in line with data from synthetic water samples as depicted in Table 4. Values were lowest for the WWTP effluent, the sample with the highest initial dissolved organic carbon, with a leaching of 0.07% (5.2 mg L^−1^ TOC) for [P_66614_][HNA]. Salinity led to decreased stability for [N_1888_][HNA] and [P_1888_][HNA], but to increased stability for [P_66614_][HNA], as observed earlier for the synthetic water samples. We conclude that the three ILs can preserve the excellent, low leaching behavior even in natural sample matrices.

## Conclusion

The three novel ILs presented in this work could be produced easily, reproducibly as well as in high purity and yield. They were capable of extracting the toxic heavy metals Ag, Cd, Cu, and Pb from “pure water” samples, with especially high efficacies for Cu and Pb. The presence of humic acids decreased Pb and Cu extraction values. NaCl in seawater-like concentrations increased the extraction of Ag and Cd. The overall best efficacies for the four metals were achieved for spiked drinking water. This supports the possibility of developing further applications in natural freshwater. We also investigated those differences in extraction behavior and leaching attributed to the central atom or alkyl chain length of the applied IL. The ammonium IL showed an increased extraction speed as well as higher stability compared to the phosphonium IL, yet was more susceptible to the negative effects on extraction efficacy caused by the different sample matrices. Comparably low leaching values were recorded in all experiments. The more hydrophobic anion chosen in this study ensured a higher stability in aqueous phases during extraction, compared to similar TSILs using less hydrophobic anions.

The developed ILs showed a high selectivity toward heavy metals combined with an increased stability during extraction in aqueous solutions. This reduced leaching represents a positive development toward a greener extraction of heavy metals using ILs. They are therefore suited as metal extractants for future applications, especially those involving natural water. Future tasks will involve evaluating their toxicity on aquatic organisms and their immobilization on solid supports for the micro-extraction of heavy metals.

## Author contributions

JL-L, FJ, BK, and CM contributed to the conception and design of the study; WK contributed to synthesis and NMR analyses; PP conducted synthesis, extraction experiments, and wrote the first draft of the manuscript. All authors contributed to manuscript revision, read and approved the submitted version.

### Conflict of interest statement

The authors declare that the research was conducted in the absence of any commercial or financial relationships that could be construed as a potential conflict of interest.

## Supplementary material

Detailed synthesis protocols, relevant peaks and bands from measured spectra, additional figures on extraction efficacy as well as the temperature-dependent viscosity of [P_66614_][HNA] are included in the Supplementary Information.
