# Supplementary material for: Novel 3-Hydroxy-2-Naphthoate-Based Task-Specific Ionic Liquids for an Efficient Extraction of Heavy Metals
Source: Front Chem. 2018 May 23;6:172. doi: 10.3389/fchem.2018.00172 (PMC5974967; doi:10.3389/fchem.2018.00172)
Supplement: Supplementary file 1 [file Data_Sheet_1.DOCX]

**Novel 3-hydroxy-2-naphthoate-based task-specific ionic liquids for an efficient extraction of heavy metals**

Philip Pirkwieser^1,2^, José A. López-López^2^, Wolfgang Kandioller^1^, Bernhard K. Keppler^1^, Carlos Moreno^2^ and Franz Jirsa^1,3*^

*^1^Institute of Inorganic Chemistry, Faculty of Chemistry, University of Vienna, Waehringer Strasse 42, 1090 Vienna, Austria*

*^2^Department of Analytical Chemistry, Faculty of Marine and Environmental Sciences, University of Cádiz, PC 11510, Puerto Real, Spain*

*^3^Department of Zoology, University of Johannesburg, PO Box 524, Auckland Park, 2006, Johannesburg, South Africa*

*Correspondence:

Franz Jirsa

franz.jirsa@univie.ac.at

Supplementary information

Table of contents

*Synthesis and spectra evaluation of 3-hydroxy-2-naphthoate-based ionic liquids*

*Table S1: Elemental analysis and determination of the chloride content of [P_66614_][HNA] in %.*

*Table S2: Miscibility of the ILs with selected organic solvents*

*Figure S4: Viscosity measurement of [P_66614_][HNA] between 293 and 323K*

*Figure S5: Extraction efficacy of [P_1888_][HNA] and [N_1888_][HNA]in synthetic water samples*

*Synthesis and spectra evaluation of 3-hydroxy-2-naphthoate-based ionic liquids*

**Trihexyltetradecylphosphonium 3-hydroxy-2-napthoate, [P_66614_][HNA]**

3-Hydroxy-2-napthoic acid (3.00 g, 1 eq., 15.9 mmol) was transferred to a round-bottom flask and dissolved in methanol (50 mL). KOH (1.07 g, 1.2 eq, 19.1 mmol) was added and the reaction mixture was stirred for several minutes. Then, Cyphos^®^ IL 101 (8.25 g, 1 eq., 15.9 mmol) dissolved in methanol (30 mL) was added. The reaction mixture was stirred for 3 hours at 40°C. Finally, methanol was removed under reduced pressure and the residue was extracted with dichloromethane/water (50 mL/30 mL) and washed four times with 30 mL water. The separated organic layer was dried over anhydrous Na_2_SO_4_, filtered, concentrated under reduced pressure and dried *in* *vacuo*. Yield: 95%, dark orange viscous oil. ^1^H NMR (500.10 MHz, CDCl_3_): *δ* = 8.56 (s, -OH), 7.75 (d, ^3^*J* = 7 Hz, 1H, H_arom_), 7.60 (d, ^3^*J* = 8 Hz, 1H, H_arom_), 7.37–7.32 (m, 4H, H_arom_), 7.20–7.16 (m, 1H, H_arom_), 7.15 (dd, ^3^*J* = 7 Hz, ^3^*J* = 7 Hz, 1H, H_arom_), 2.32–2.17 (m, 8H, –CH_2_–), 1.52–1.35 (m, 17H, –CH_2_–), 1.34–1.13 (m, 36H, –CH_2_–), 0.92–0.77 ppm (m, 12H, –CH_3_). IR (ATR, selected bands, ν_max_): 2925, 2857, 1652, 1577, 1520, 1454, 1365, 1327, 1226, 864, 779, 742, 594 cm^−1^.


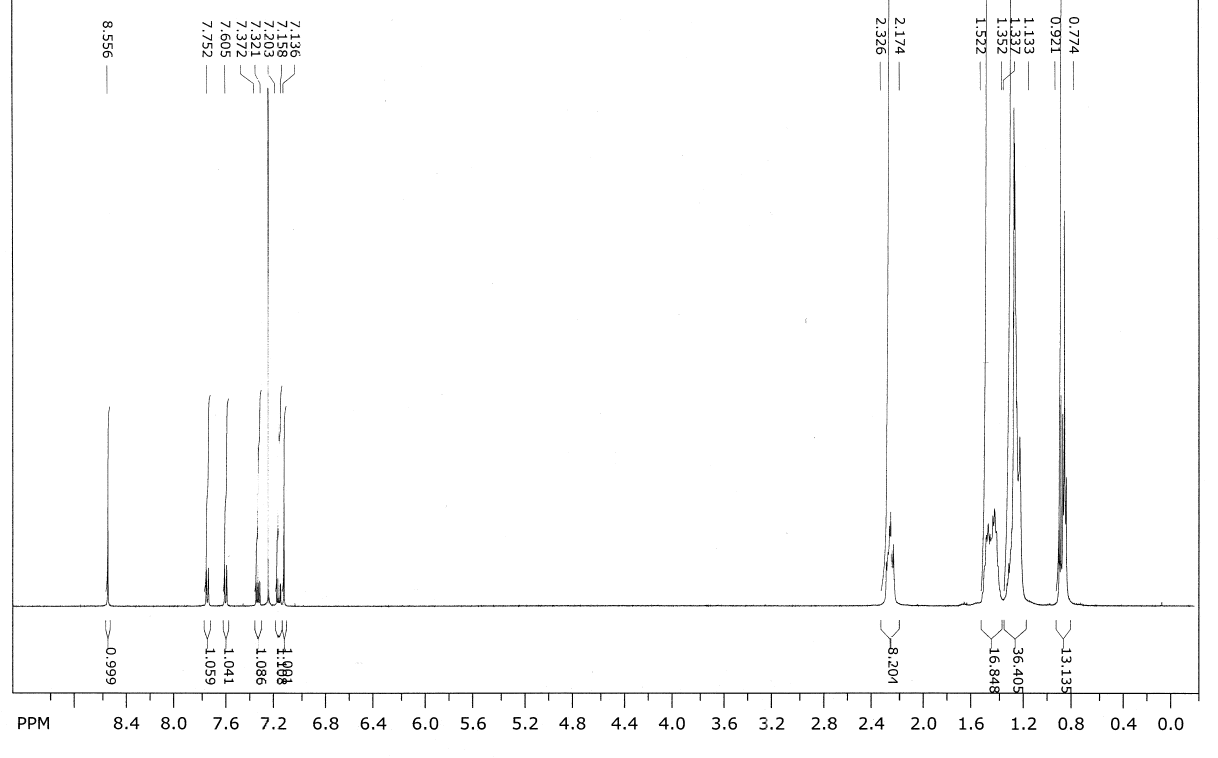


Figure S1: ^1^H-NMR spectrum of IL [P_66614_][HNA].

**Methyltrioctylphosphonium 3-hydroxy-2-naphthoate, [P_1888_][HNA]**

3-Hydroxy-2-napthoic acid (3.00 g, 1 eq., 15.9 mmol) was transferred to a round-bottom flask and dissolved in methanol (50 mL). Methyltrioctylphosphonium methylcarbonate (59.8% in methanol, 12.25 g, 1 eq, 15.9 mmol) was added and the solution stirred for 1 hour. The solvent was removed under reduced pressure and the obtained IL was dried *in vacuo* at 50 °C overnight. Yield: 100%, orange solid. ^1^H NMR (500.10 MHz, CDCl_3_): *δ* 8.51 (s, -OH), 7.74 (d, ^3^*J* = 7 Hz, 1H, H_arom_), 7.59 (d, ^3^*J* = 8 Hz, 1H, H_arom_), 7.34 (dd, ^3^*J* = 8 Hz, ^3^*J* = 8 Hz, 4H, H_arom_), 7.17 (dd, ^3^*J* = 7 Hz, ^3^*J* = 7 Hz, 1H, H_arom_), 7.13 (dd, ^3^*J* = 7 Hz, ^3^*J* = 7 Hz, 1H, H_arom_), 2.27–2.14 (m, 6H, –CH_2_–), 1.95 (m, 3H, –CH_3_), 1.50–1.32 (m, 14H, –CH_2_–), 1.31–1.12 (m, 36H, –CH_2_–), 0.91–0.79 ppm (m, 12H, –CH_3_). IR (ATR, selected bands, ν_max_): 2924, 2855, 1652, 1623, 1578, 1521, 1454, 1365, 1222, 838, 781, 742, 594 cm^−1^.


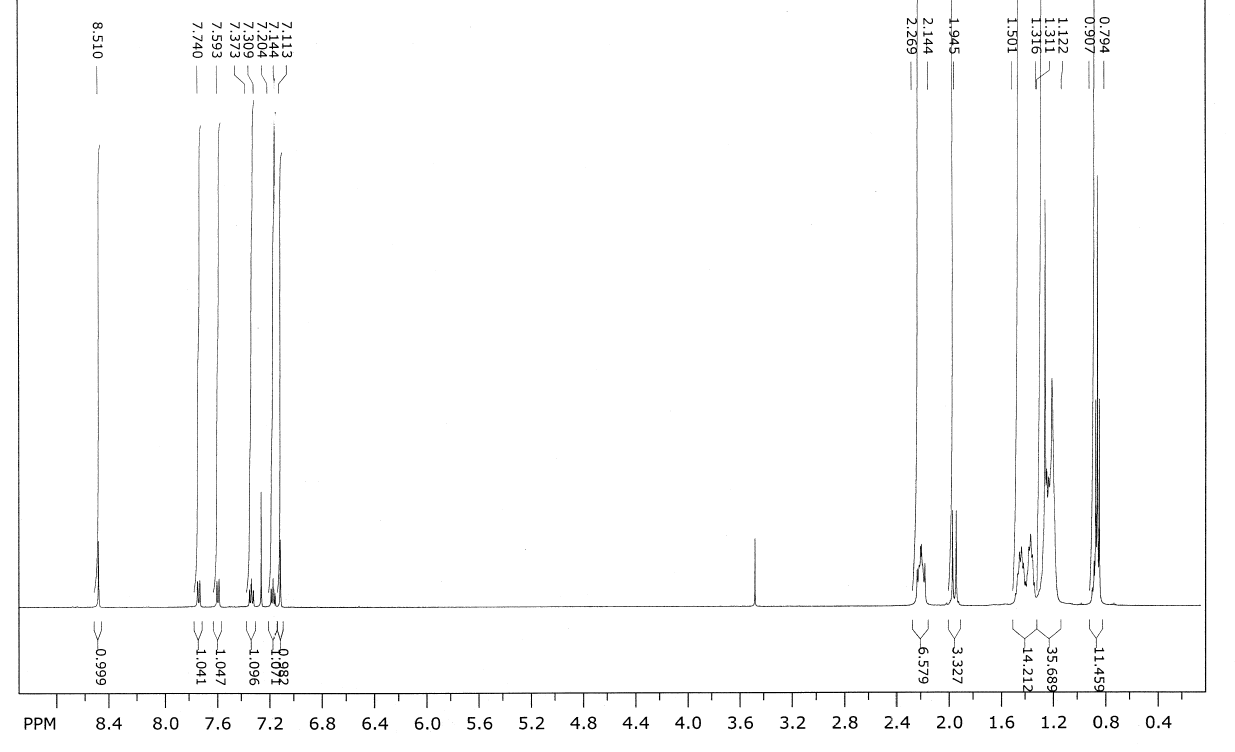


Figure S2: ^1^H-NMR spectrum of IL [P_1888_][HNA].

**Methyltrioctylammonium 3-hydroxy-2-napthoate, [N_1888_][HNA]**

3-Hydroxy-2-napthoic acid (3.00 g, 1 eq., 15.9 mmol) was transferred to a round-bottom flask and dissolved in methanol (50 mL). Methyltrioctylammonium methylcarbonate (48.6% in methanol, 14.56 g, 1 eq., 15.9 mmol) was added and the solution was stirred for 1 hour. The solvent was removed under reduced pressure and the obtained IL was dried *in vacuo* at 50 °C overnight. Yield: 100%, yellow-orange solid. ^1^H NMR (500.10 MHz, CDCl_3_): *δ* 8.53 (s, ‑OH), 7.75 (d, ^3^*J* = 7 Hz, 1H, H_arom_), 7.60 (d, ^3^*J* = 8 Hz, 1H, H_arom_), 7.34 (dd, ^3^*J* = 8 Hz, ^3^*J* = 8 Hz, 4H, H_arom_), 7.20–7.14 (m, 1H, H_arom_), 7.13 (dd, ^3^*J* = 7 Hz, ^3^*J* = 7 Hz, 1H, H_arom_), 3.34–3.18 (m, 9H, –CH_2_–), 1.65–1.52 (m, 7H, –CH_2_–), 1.35–1.11 (m, 45H, –CH_2_–), 0.90–0.79 ppm (m, 11H, –CH_3_). IR (ATR, selected bands, ν_max_): 2924, 2856, 1651, 1622, 1580, 1523, 1453, 1366, 1223, 837, 780, 741, 594 cm^−1^.


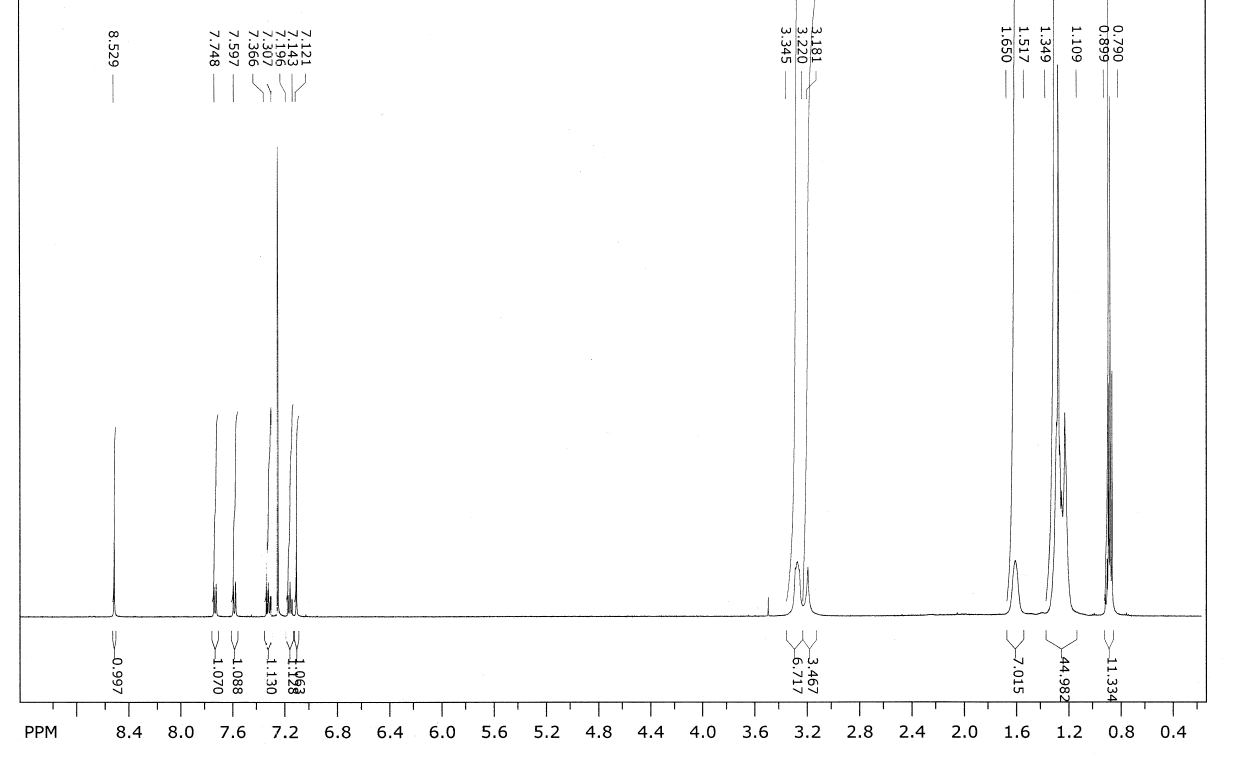


Figure S3: ^1^H-NMR spectrum of IL [N_1888_][HNA].

|  | Mass fraction (wt%) | | | | | |
| --- | --- | --- | --- | --- | --- | --- |
|  | C | H | N | S | O | Cl |
| Calculated | 76.97 | 11.27 | - | - | 7.15 | - |
| Sample 1 | 75.84 | 11.24 | <0.05 | <0.02 | 7.73 | 0.175 |
| Sample 2 | 76.08 | 11.35 | <0.05 | <0.02 | 8.08 | 0.125 |

Table S1: Elemental analysis and determination of the chloride content of [P_66614_][HNA] in %.

| Ionic liquid | H_2_O | MeOH | EtOH | Ethyl  acetate | Diethyl  ether | Acetone | *n*-hexane | CH_2_Cl_2_ | Aceto  nitrile | Tetra  hydro  furane |
| --- | --- | --- | --- | --- | --- | --- | --- | --- | --- | --- |
| [P_66614_][HNA] | 🗶 | ✓ | ✓ | ✓ | ✓ | ✓ | 🗶 | ✓ | ✓ | ✓ |
| [P_1888_][HNA] | 🗶 | ✓ | ✓ | ✓ | ✓ | ✓ | 🗶 | ✓ | ✓ | ✓ |
| [N_1888_][HNA] | 🗶 | ✓ | ✓ | ✓ | 🗶 | ✓ | 🗶 | ✓ | ✓ | ✓ |

Table S2: Miscibility of the ILs with selected organic solvents, determined visually by adding the respective solvent to small amounts of each IL.


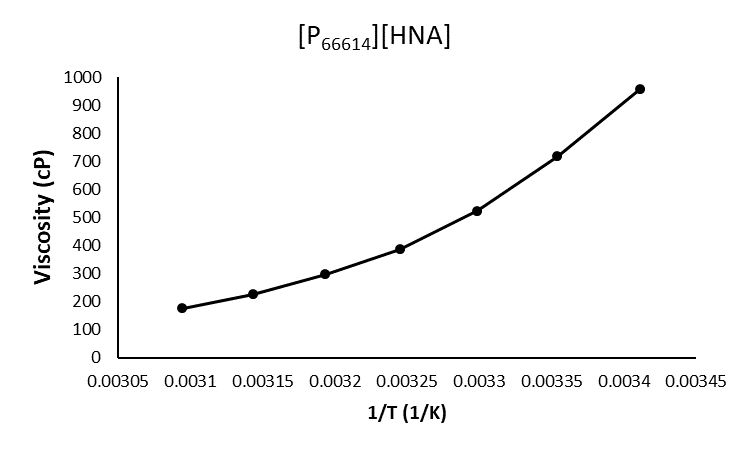


Figure S4: Viscosity measurement of [P_66614_][HNA] between 293 and 323K.


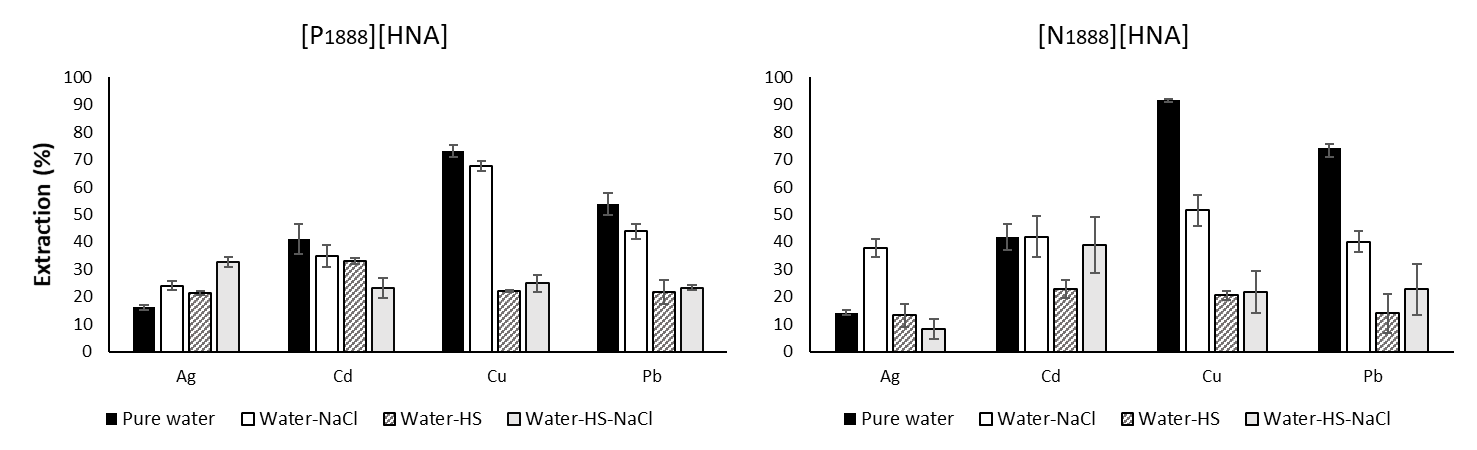


Figure S5: Extraction efficacies in synthetic water samples using 300 mg of [P_1888_][HNA] or [N_1888_][HNA] in 30 mL of the respective feed solution for an extraction time of 1 h. n=3, error bars = ± SD.
